# Serum miR-143 levels predict the pathological response to neoadjuvant chemoradiotherapy in patients with locally advanced rectal cancer

**DOI:** 10.18632/oncotarget.16760

**Published:** 2017-03-31

**Authors:** Yukiharu Hiyoshi, Takashi Akiyoshi, Ramu Inoue, Keiko Murofushi, Noriko Yamamoto, Yosuke Fukunaga, Masashi Ueno, Hideo Baba, Seiichi Mori, Toshiharu Yamaguchi

**Affiliations:** ^1^ Gastroenterological Center, Department of Gastroenterological Surgery, The Cancer Institute Hospital of Japanese Foundation for Cancer Research, Tokyo, Japan; ^2^ Clinical Research Center, The Cancer Institute Hospital of Japanese Foundation for Cancer Research, Tokyo, Japan; ^3^ Department of Radiation Oncology, The Cancer Institute Hospital of Japanese Foundation for Cancer Research, Tokyo, Japan; ^4^ Division of Pathology, The Cancer Institute Hospital of Japanese Foundation for Cancer Research, Tokyo, Japan; ^5^ Department of Gastroenterological Surgery, Graduate School of Medical Sciences, Kumamoto University, Kumamoto, Japan; ^6^ Division of Cancer Genomics, The Cancer Institute Hospital of Japanese Foundation for Cancer Research, Tokyo, Japan

**Keywords:** rectal cancer, chemoradiotherapy, serum, miR-143, prediction

## Abstract

Recently, several circulating miRNAs have been reported as promising, minimally invasive biomarkers for the diagnosis or prediction of the prognosis in various types of cancer. However, the utility of circulating miRNAs as predictive markers of the cancer response to neoadjuvant chemoradiotherapy (nCRT) for locally advanced rectal cancer is still unclear. To identify circulating serum miRNAs useful for predicting a pathological good response to nCRT, total 18 serum miRNAs of interest were analyzed by real-time polymerase chain reaction in 94 rectal cancer patients treated with nCRT and surgery. Pathological complete response (pCR; Dworak TRG4) and near-pCR (TRG3) were obtained in 12 (13%) and 9 (9%) patients respectively, and we regarded them as nCRT-responders. Of the 18 serum miRNAs, only the serum level of miR-143 was identified significantly associated with a pathological response to nCRT in 94 patients; the serum miR-143 level was significantly lower in nCRT-responders than in non-responders. A multivariate analysis incorporating other clinicopathological factors showed that only the serum miR-143 level was an independent predictor of a good pathological response. The circulating serum miR-143 level may be a novel, non-invasive predictive marker of a response to nCRT in locally advanced rectal cancer patients.

## INTRODUCTION

Colorectal cancer is one of the main causes of cancer mortality worldwide. Approximately 30% of colorectal cancer cases are designated as rectal cancer, and approximately 40% of these are locally advanced at the diagnosis [1]. The therapeutic management of locally advanced rectal cancer has drastically changed in the last 40 years, and in contrast to colon cancer, it typically involves pre-operative neoadjuvant chemoradiotherapy (nCRT) followed by surgery [2]. The response to nCRT varies from none to complete [3, 4] and only the responding patients seem to experience a good outcome [5, 6]. To avoid the unnecessary exposure to toxic and inefficient therapies in non-responder patients and quickly proceed with surgery, the identification of reliable and non-invasive predictive markers of the cancer response to nCRT is crucial. Although many clinical, metabolic, biological and imaging tools have been evaluated as predictive markers, the performance of these methods for predicting the pathological response is poor [7–12].

MicroRNAs (miRNAs) are a class of small, non-coding RNAs ranging in size from 19 to 25 nucleotides and act as post-transcriptional regulators of gene expression by silencing their ′-UTR-mRNA targets [13, 14]. Aberrant miRNA expression has been consistently reported in tissues of various human malignancies [15], and some miRNAs in rectal cancer tissues have been associated with the tumor response to nCRT [16–21]. As some of these dysregulated miRNAs are secreted into the blood, and circulating cell-free miRNAs can be detected in serum or plasma in highly stable forms, circulating miRNAs have emerged as potential blood-based biomarkers for human cancer [22]. Because miRNAs are involved in many cellular pathways, a proportion of circulating miRNAs are likely derived from the tumor microenvironment as well as blood cells and other tissues [22]. Although several circulating miRNAs have been reported as promising, minimally invasive biomarkers for colorectal cancer [23], the utility of these miRNAs as predictive markers of the cancer response to nCRT is still unclear.

The aim of this study was to identify circulating miRNAs in serum predicting the pathological response to nCRT in patients with locally advanced rectal cancer. We first selected candidate miRNAs that had been reported to be dysregulated in cancer tissue or serum/plasma in patients with colorectal cancer [23–27]. In addition, we also selected miRNAs that have been recently reported to be associated with the response to chemotherapy or chemoradiotherapy for colorectal cancer [28–33]. A total of 18 candidate miRNAs (let-7b, miR-15b, -20a, -21, -29a, -92a, -122, -125b, -141, -143, -145, -155, -200c, -221, -345, -423, -425, -1246) were selected, and their serum levels obtained from rectal cancer patients before nCRT were quantified by TaqMan real-time polymerase chain reaction (PCR). The correlation between the pathological response to nCRT and serum miRNA level was then analyzed. In addition, not only the serum miRNA levels but also the clinicopathological factors were analyzed. To our knowledge, this is the first study evaluating the usefulness of both serum miRNA levels and clinicopathological factors as predictive markers of the response to nCRT in patients with locally advanced rectal cancer.

## RESULTS

The clinicopathological characteristics and surgical factors of 94 patients enrolled in the present study are summarized in Table 1. All clinical characteristics were evaluated before nCRT. Histological type and KRAS status were evaluated using biopsy samples obtained before nCRT. The mutation rate of KRAS gene was 43.5% (40/92). Although 7 of 94 patients had tumors with direct invasion to surrounding organs (cT4b; levator ani muscle: 3, prostate: 1, seminal vesicle: 1, vagina: 1, neurovascular bundle: 1), all resections were performed without exposure of the tumor. Clinically, 51 patients (54.3%) had suspected lymph node metastasis (29 patients in the mesorectum, 12 patients in the lateral pelvic lymph node [LPLN], and 10 patients in both) before CRT. The patient who was diagnosed as LPLN-positive underwent lateral pelvic lymph node dissection (unilateral: 20 patients, bilateral: 2 patients). In addition, synchronous liver metastases appeared in 2 patients during CRT while they were not detected in images before CRT. For these 2 patients, simultaneous liver resection was performed. Therefore, all patients underwent surgically curative resection. Pathologically, 21 patients (22.3%) had lymph node metastasis (17 patients in the mesorectum, 3 patients in the LPLN, and 1 patient in both), and 2 patients (2.1%) had liver metastases. R0 resections for all 94 patients were confirmed by a pathological examination.

**Table 1:** Clinicopathological characteristics and surgical factors of patients

Factor	Variable	Value
Age (years)	mean (range)	58.7 (27–79)
Gender	Male/Female	63/31
AV-tumor distance (mm)	mean (range)	45.3 (5–85)
Tumor size (mm)	mean (range)	37.0 (16–94)
Histological type	well/mod/pap/por	62/29/1/2
CEA	negative/positive	69/25
CA19-9	negative/positive	88/6
KRAS status	wild type/mutant/unknown	52/40/2
cT^1^	2/3/4a/4b	1/85/1/7
cN^1^	0/1/2	43/39/12
CRT-ope (days)	mean (range)	50.1 (19–112)
Operation^2^	LAR	38
	ISR	29
	Hartmann’s operation	1
	APR	26
LPND^2^	none/unilateral/bilateral	72/20/2
pT^1^	pCR/ is /1/2/3/4b	12/1/7/30/42/2
pN^1^	0/1/2	73/20/1
pM^1^	0/1a	92/2

AV: anal verge, CRT: chemoradiotherapy, LAR: low anterior resection, ISR: intersphincteric resection, APR: abdominoperineal resection, LPND: lateral pelvic node dissection, well: well differentiated adenocarcinoma, mod: moderately differentiated adenocarcinoma, pap: papillary adenocarcinoma, por: poorly differentiated adenocarcinoma

^1^The clinical and pathological findings were classified according to the TNM classification (7th edition) of the International Union against Cancer (UICC).

^2^All surgical treatments were performed laparoscopically.

**Figure 1:**
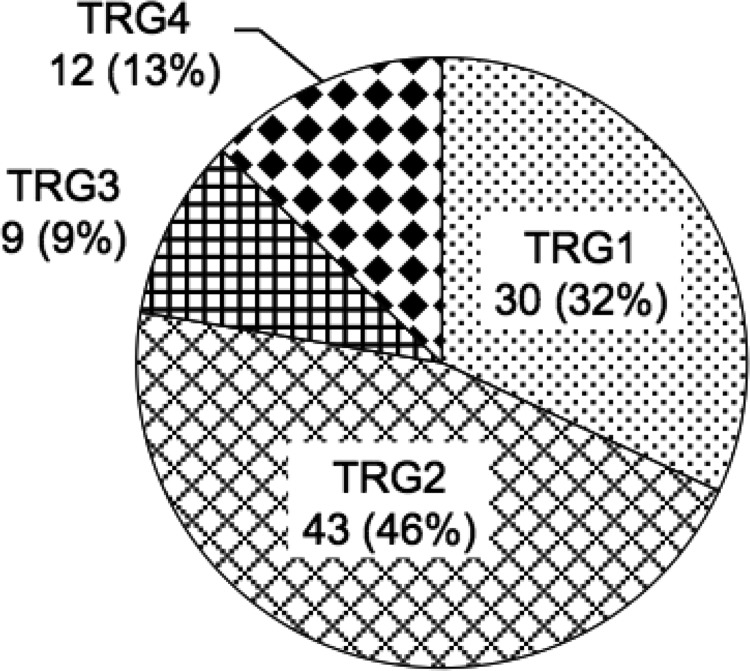
The pathological response to nCRT The pathological response to nCRT was determined by a histopathological examination of surgically resected specimens based on the Dworak tumor regression grading (TRG) system. The extent of histopathologic regression was divided into five categories: TRG0 (No regression), TRG1 (Dominant tumor mass with obvious fibrosis and/or vasculopathy), TRG2 (Dominant fibrotic changes with few tumor cells or groups), TRG3 (Very few tumor cells in fibrotic tissue with or without mucus substance), and TRG4 (No tumor cells, only fibrotic mass; pathological complete response, pCR). No patient had TRG0 tumor.

Figure 1 shows the pathological response to nCRT evaluated in surgically resected primary lesions. The pathological responses were as follows: Dworak TRG4 (pathological complete response; pCR) in 12 patients (13%), TRG3 (near pCR) in 9 patients (9%), TRG2 in 43 patients (46%), TRG1 in 30 patients (32%) and no TRG0 tumor. We defined patients with grade TRG4 or TRG3 tumor as responders, and those with TRG2 or TRG1 tumor as non-responders.

**Figure 2:**
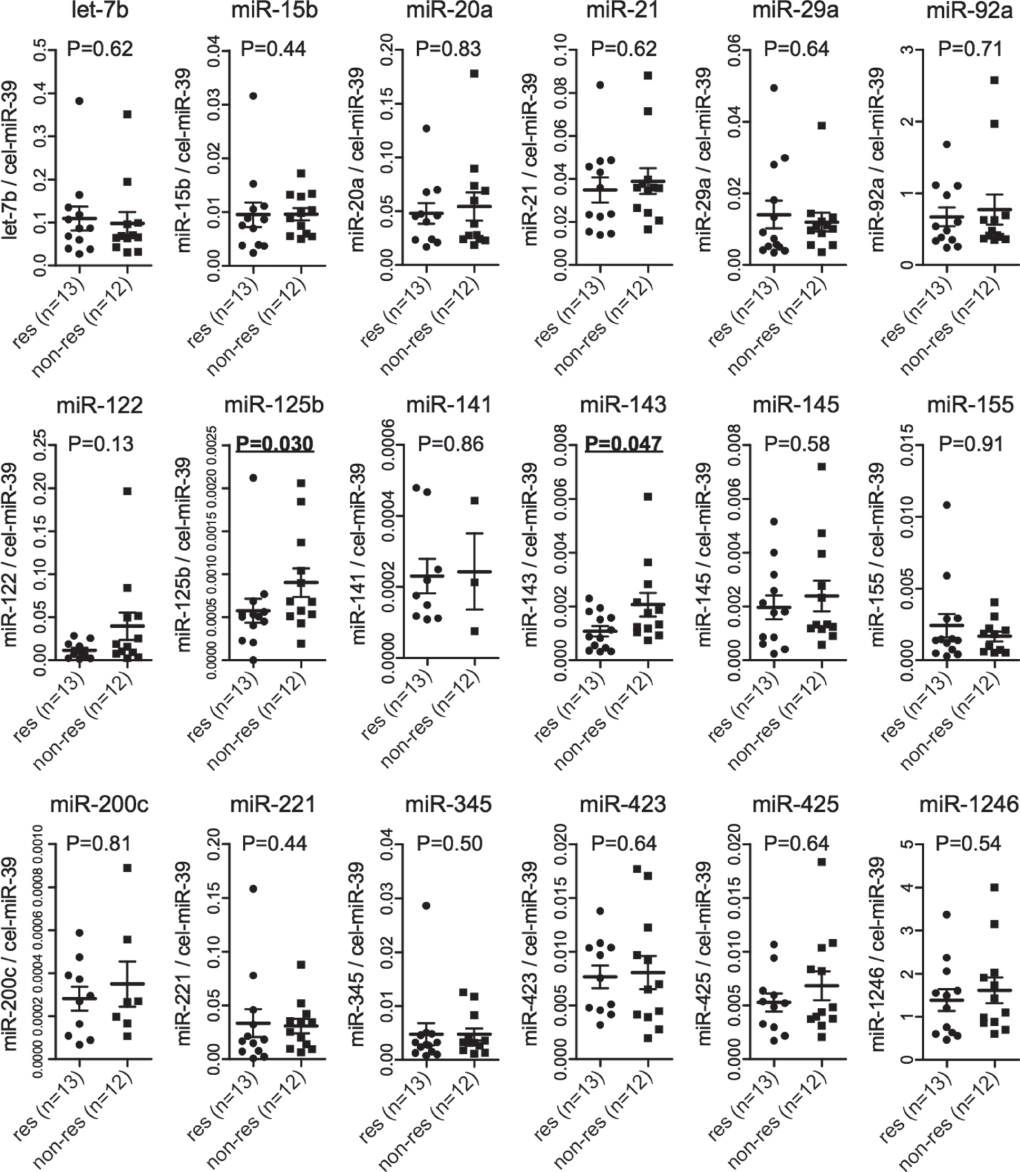
Serum miRNA levels in 25 patients At first, we selected the 13 patients with the best response (12 pCR and 1 near-pCR) as responders and the 12 patients with the poorest response (only TRG1) as non-responders in order to identify clear differences in the serum miRNA levels between responders and non-responders. The dot plots represent 18 miRNA levels quantified by TaqMan real-time PCR and normalized to external controls: cel-miR-39 level using ΔΔCt method stratified by the pathological response to nCRT. The horizontal bars indicate the median value and 95% confident interval. The Mann Whitney-U test was used. Res (responder): TRG4 and TRG3. Non-res (non-responder): TRG2 and TRG1. NS: not significant.

To identify the serum miRNAs associated with the pathological response to nCRT as predictive marker, we analyzed 18 miRNAs (let-7b, miR-15b, -20a, -21, -29a, -92a, -122, -125b, -141, -143, -145, -155, -200c, -221, -345, -423, -425, -1246) via TaqMan real-time PCR using serum obtained from patients before nCRT. We first conducted our analysis using a small number of samples. We selected the 13 patients with the best response (12 pCR and 1 near-pCR) as responders and the 12 patients with the poorest response (only TRG1) as non-responders in order to identify clear differences in the serum miRNA levels between responders and non-responders. Figure 2 shows the comparison of the 18 serum miRNAs between responders and non-responders. Of the 18 miRNAs, miR-125b and miR-143 showed significant differences between responders and non-responders with both miR-125b and miR-143 levels in serum being significantly lower in responders than in non-responders (*P* = 0.030 and 0.047, respectively). In addition, the serum miR-122 level was also lower in responders than in non-responders, but not to a significant degree (*P* = 0.13). To confirm the reproducibility of these findings, we repeated RNA extraction and real-time PCR for a serum miRNA analysis in the same 25 patients. The Pearson correlation coefficients for the first and second analyses showed a very strong correlation with the serum miR-143 level (Supplementary Figure 1, r = 0.9140, *P* < 0.0001).

**Figure 3:**
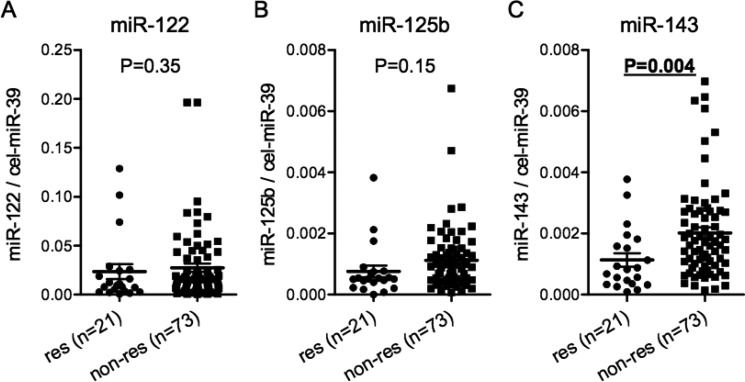
Serum miR-143 predicts the pathological response to nCRT in all 94 patients.Comparison of the serum levels of miR-122 (A), miR-125b (B) and miR-143 (C) normalized to cel-miR-39 between responders and non-responders in all 94 patients The dot plots represent serum miRNA levels quantified by TaqMan real-time PCR and normalized to external controls: cel-miR-39 level using ΔΔCt method. The horizontal bars indicate the median value and 95% confident interval. The Mann Whitney-U test was used. Res (responder): TRG4 and TRG3. Non-res (non-responder): TRG2 and TRG1.

We then analyzed the serum miR-122, miR-125b and miR-143 levels in all 94 patients to confirm the findings obtained in Figure 2. The serum miR-143 level was also significantly lower in responders than in non-responders. (Figure 3C, *P* = 0.004). The finding was similar when patients were stratified into TRG4 and TRG1-3 groups (Supplementary Figure 2). However, there was no significant difference in the serum miR-122 and miR-125b levels between responders and non-responders (Figure 3A and 3B).

To investigate the serum miR-143 levels in healthy controls, we collected serum samples from 12 healthy volunteers. The serum miR-143 levels of healthy controls were significantly higher than those of CRT responders or non-responders (Supplementary Figure 3, *P* < 0.0001). In addition, the serum miR-143 levels were compared between pre- and post-nCRT (just before surgery) using available serum samples from 76 of 94 patients (18 responders and 58 non-responders) (Supplementary Figure 4). In these 76 patients, the serum miR-143 levels after CRT were significantly lower than before CRT. Interestingly, a significant change in the serum miR-143 was observed only in CRT non-responders, and the miR-143 level did not change markedly in CRT responders.

**Figure 4:**
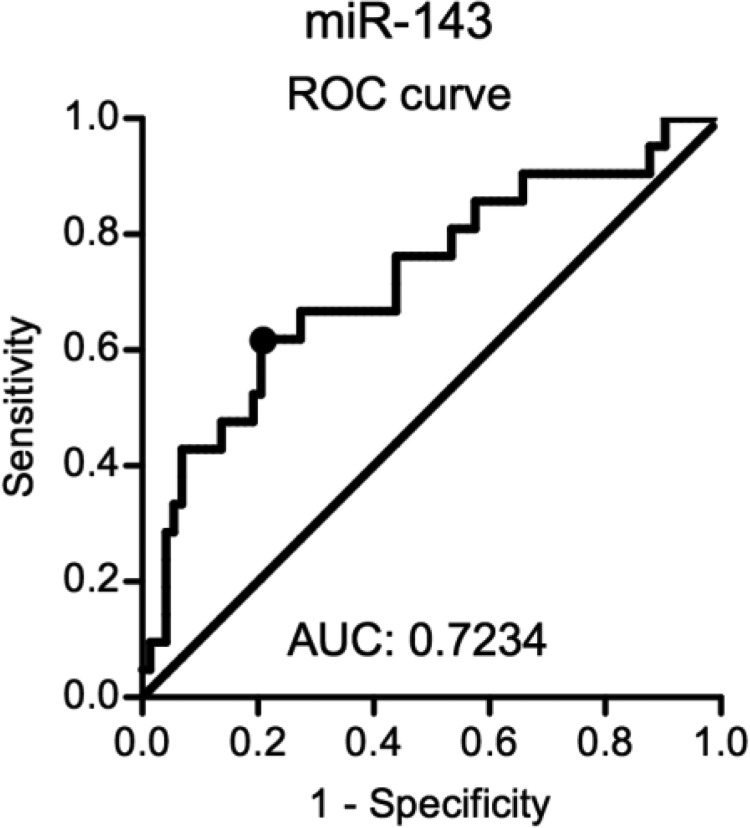
An ROC analysis for the serum miR-143 level The cut-off of serum miR-143 level was determined using the Youden index. ROC: receiver operating characteristic, AUC: area under the curve.

Figure 4 shows a receiver operating characteristic (ROC) analysis of the pathological response for serum miR-143. The area under curve (AUC) was 0.7234, and the cut-off for the serum miR-143 level (0.00093) was established using the Youden index. With this cut-off point the sensitivity and specificity were 0.6190 and 0.7945, respectively. The serum miR-143 levels in 2 patients who had synchronous liver metastasis at surgery were 0.00025 and 0.00087, and both were classified as low by our cut-off line.

Table 2 shows the results of the univariate and multivariate analyses for identifying the factors associated with the pathological response to nCRT. Among the analyzed factors listed in Table 2, only the serum miR-143 level was significantly associated with the pathological response in the univariate analysis; specifically, a low level of serum miR-143 was significantly associated with good pathological response (*P* < 0.001). In the multivariate analysis incorporating the factors for which the *p*-value in the univariate analysis was less than 0.2, a low level of serum miR-143 remained an independent predictor of a good pathological response (HR: 0.173, 95% CI: 0.060–0.500, *P* = 0.001). As shown in Supplementary Table 1, no clinicopathological findings showed a significant association with the serum miR-143 level.

**Table 2:** Factors associated with pathological response to preoperative CRT

Factor		Responder (*n =* 21)	Non-responder (*n =* 73)	*P*-value	Multivariate analysis	
					HR (95% CI)	*P*-value
Age (years)	≤ 60/> 61	7/14	39/34	0.105	1.831 (0.617–5.438)	0.276
Gender	Male/Female	14/7	49/24	0.969		
AV-tumor distance (mm)	≤ 40/> 41	13/8	38/35	0.425		
Tumor size (mm)^1^	≤40/>40	12/9	44/27	0.690		
Histological type	well, mod, pap/por	20/1	72/1	0.399		
CEA	negative/positive	17/4	52/21	0.576		
CA19-9	negative/positive	21/0	67/6	0.332		
KRAS^2^	wild type/mutant	10/9	42/31	0.701		
cT^3,4^	2, 3/4	21/0	65/8	0.192		
cN^3^	0/1, 2	11/10	32/41	0.488		
CRT-ope (days)	≤ 50/> 51	14/7	42/31	0.452		
Serum miR-143^5^	low/high	13/8	15/58	< 0.001	0.173 (0.060–0.500)	0.001

^1^The tumor size before CRT was not evaluated by enema examination in two cases.

^2^The KRAS status in two cases was not evaluated.

^3^The clinical findings were classified according to the TNM classification (7th edition) of the International Union against Cancer (UICC).

^4^cT was excluded from the multivariate analysis because there were no patients with cT4 tumors in the responder group and the estimated HR value was indefinite.

^5^The cut-off for the serum miR-143 level (0.00093) was established by an ROC analysis.

## DISCUSSION

In the present study, we first selected 18 miRNAs that had been reported to be dysregulated in cancer tissues or serum as well as associated with the tumor response to multimodal treatments, such as chemotherapy and/or radiotherapy, in colorectal cancer patients. A real-time PCR analysis using serum samples showed that only serum miR-143 level was significantly associated with the pathological response to nCRT in locally advanced rectal cancer. In addition, a multivariate analysis incorporating clinicopathological factors showed that only a low serum miR-143 level was an independent predictor of a good pathological response. Although the prognostic value of serum miR-143 could not be analyzed because the postoperative follow-up period of most patients enrolled in the present study was short, these findings suggest that serum miR-143 may be a novel, non-invasive predictive marker of the response to nCRT in rectal cancer patients.

The accurate prediction of the pathological response to nCRT in locally advanced rectal cancer before treatment is crucial for determining the therapeutic strategy. Previous studies have shown that rectal cancer patients with a pathological good response after nCRT achieved favorable outcomes [5, 6]. In addition, recent studies have shown that tumor regression would prevent patients from surgery, that is called “watch and wait” approach [34, 35]. However, radioresistant tumors may need dose-intensive chemotherapy to improve the therapeutic outcomes. In the present study, a pathological good response (pCR and near-pCR) was achieved in about 20% of patients, and the response rate was consistent with that shown in previous reports [36–38]. Several previous reports have tried to identify predictive markers of response to nCRT, with some finding that pCR was associated with good tumor differentiation, a small tumor diameter, early T and N stage on imaging, low levels of pretreatment CEA and KRAS mutations [39]. Therefore, we focused on these factors as well as the serum miRNA level. However, none of these clinicopathological factors subjected to our multivariate analysis was found to be a predictive marker of the pathological response.

miRNA expression analyses using rectal cancer tissues comparing between CRT-responders and non-responders have revealed predictive markers of a therapeutic response [16–21]. However, predicting the tumor response to nCRT using a tissue analysis requires obtaining cancer tissues by a biopsy prior to nCRT. A less-invasive alternative, several circulating miRNAs have been reported as promising diagnostic biomarkers for colorectal cancer [23]. Indeed, two recent studies have shown the potential utility of circulating miRNAs as predictive markers for the response to preoperative treatments in rectal cancer patients. D’Angelo et al. found that a high level of miR-125b in cancer tissue and serum was associated with a poor response to nCRT in patients with locally advanced rectal cancer, suggesting that miR-125b could be a novel non-invasive biomarker of response [29]. Similarly, Yu et al. showed that high miR-345 expression in cancer tissue and serum was significantly correlated with an unfavorable pathological response to nCRT, and a low level of serum miR-345 predicted a superior three-year local recurrence-free survival [31]. In the present study, these 2 miRNAs were included in the 18 miRNAs of interest. However, while a high level of miR-125b was associated with a poor response to nCRT in the first set of 25 patients, this finding was not validated when we conducted an analysis in all 94 patients. No significant association was noted between serum miR-345 levels and the pathological response in the present study.

miR-143 located on chromosome 5q33 in the human genome is a well-known tumor suppressive miRNA in various types of malignancies, including colorectal cancer. The downregulation of miR-143 in both colorectal cancer tissues and cell lines has been reported, and the abnormal expression of miR-143 can regulate cell proliferation, cell growth, clone formation, apoptosis, cell cycle, invasion and migration by targeting a number of genes including COX-2, MMP-13, GLI3, DNMT3A and KRAS [40–42]. The association between the miR-143 expression in colorectal cancer tissues and sensitivity to chemotherapy has been investigated in several studies. Consistent with its tumor-suppressive features, Borralho et al. [43] and Qian et al. [44] showed that miR-143 increased the chemosensitivity of colorectal cancer cells to 5-fluorouracil and oxaliplatin treatment respectively, in *in vitro* experiment. However, little is known about the association between miR-143 and the response to chemoradiotherapy in patients with locally advanced rectal cancer.

Generally, as a result of apoptotic and necrotic cell death, miRNAs are released into the circulation, and the circulating cell-free miRNAs in the bloodstream are highly stable [22]. Because miRNAs are involved in many cellular pathways, a proportion of circulating miRNAs are likely derived from various types of cells including tumor cells. Kent et al. found miR-143 to be among the most abundant miRNAs in mesenchymal cells, including fibroblasts and smooth muscle cells, with levels being significantly lower in other cell types, including epithelial cells, endothelial cells and red blood cells [45]. In mesenchymal cells, miR-143 has been shown to play a role in cellular differentiation (e.g. maturing adipocytes) [46]. In the present study, the serum miR-143 levels of healthy controls were significantly higher than those in CRT responders or non-responders. The high-serum miR-143 level in healthy controls seems to be derived from normal cells, such as fibroblasts and smooth muscle cells [45]. In addition to its potential as predictive marker for a response to nCRT, this finding suggests that decreased levels of serum miR-143 may also be a potential diagnostic marker of rectal cancer.

In the present study, a low pretreatment serum level of miR-143 in rectal cancer patients was associated with a good pathological response to nCRT. However, whether the circulating miR-143 in rectal cancer patients originates from tumor cells or other cells, such as mesenchymal cells, is unclear. If the serum miR-143 detected in the present study did indeed originate from cancer cells, the fact that a low level of serum miR-143 was associated with a good response may be inconsistent with the tumor-suppressive role of miR-143. Since miR-143 targets many genes, each with its own transcriptional regulation, the function of miR-143 may differ by circumstances (e.g. *in vivo* vs. *in vitro*; chemotherapy vs. chemoradiotherapy). Indeed, consistent with our findings, some recent studies have shown potential roles of miR-143 associated with resistance to chemotherapy or radiotherapy. Simmer et al. reported that patients with metastatic colorectal cancer with a low expression of miR-143 in cancer tissue had an increased median progression-free survival (PFS) after being treated with fluoropyrimidine-based chemotherapy compared to those with high expression of miR-143. They identified an ion transport regulator FXFD3 as a putative target of miR-143 affecting chemosensitivity [47]. Lin et al. showed that miR-143 protects cells from DNA damage-induced killing including ionizing radiation by targeting the fragile histidine triad (FHIT) [48]. One possible explanation regarding why serum miR-143 levels decreased after nCRT in only CRT-non-responders may be that serum miR-143 derived from CRT-resistant tumors decreased in non-responders concordant with the regression of tumors by CRT because even pathological non-responders often showed tumor shrinkage clinically. In contrast, the serum level of miR-143 may not change after CRT in responders because CRT-sensitive tumors do not innately express miR-143. Further analyses are needed to confirm the origin of serum miR-143 and its biological roles associated with resistance to nCRT.

Several limitations associated with the present study warrant mention. First, although the AUC of 0.7234 in our ROC analysis for serum miR-143 reflected good discrimination in terms of sensitivity and specificity, the power is still not enough. Identification of other predictive markers that can be used in combination with serum miR-143 might be needed for a more accurate prediction of the potential response to nCRT and development of tailored treatment strategies for rectal cancer. Second, our results should be validated in a larger prospective study for future clinical application. Third, the miR-143 expression analysis in cancer tissues or the functional analysis of miR-143 via *in vitro* experiments should be performed to elucidate the origin of the circulating miR-143 and its biological function affecting the chemoradiosensitivity.

In conclusion, serum miR-143 may be a novel, non-invasive predictive marker of the response to nCRT in patients with locally advanced rectal cancer. This finding suggests that treatment strategies for rectal cancer patients may be able to be individualized.

## MATERIALS AND METHODS

### Patients and clinical staging

In the present study, we prospectively collected serum from 94 patients with locally advanced low rectal cancer who underwent curative resection at our institution from July 2013 to May 2016 following neoadjuvant chemoradiation therapy (nCRT). The clinicopathological data were retrospectively collected from our prospectively maintained database. Low rectal cancer is defined as a tumor with an inferior border located below the peritoneal reflection, which is equivalent to 8 cm from the anal verge. Pre-treatment clinical staging according to the TNM classification (7th edition) of the International Union against Cancer (UICC) was performed using a combination of a physical examination and cross-sectional imaging with either computed tomography (CT) or magnetic resonance imaging (MRI). All patients provided their written informed consent to participate in this study. The local ethics committee of the Cancer Institute Hospital of JFCR approved this study.

### nCRT and surgery

The indications for nCRT were low rectal cancer below the peritoneal reflection; full-thickness rectal cancers (T3 or T4) staged by MRI and/or node-positive diseases; no evidence of distant metastasis; and no history of prior radiation therapy to the pelvis. All patients received nCRT with a total dose of 50.4 Gy (28 fractions of 1.8 Gy) of pelvic irradiation with concurrent oral 5-fluorouracil (S-1). Surgical treatment was performed 4–8 weeks after the completion of nCRT, and all surgeries were performed laparoscopically. If preoperative CT or MRI showed swollen lateral pelvic lymph nodes (LPLNs) (≥ 7 mm), lateral pelvic lymph node dissection for only the metastasized side was performed, regardless of the imaging after CRT. If no swollen LPLNs were detected, only standard TME was performed.

### Histopathologic response evaluation

The histopathologic response to nCRT was determined by a histopathologic examination of surgically resected specimens based on a the Dworak tumor regression grading (TRG) system [49]. The extent of histopathologic regression was divided into five categories: TRG0 (No regression), TRG1 (Dominant tumor mass with obvious fibrosis and/or vasculopathy), TRG2 (Dominant fibrotic changes with few tumor cells or groups), TRG3 (Very few tumor cells in fibrotic tissue with or without mucus substance), and TRG4 (No tumor cells, only fibrotic mass: pCR). We defined patients with TRG4 or TRG3 tumor as responders, and those with TRG2, 1, 0 tumors as non-responders.

### Serum preparation and RNA extraction

About 5 ml venous blood was collected from patients and healthy volunteers. The whole blood was separated into serum and cellular fractions by centrifugation at 3000 rpm for 10 min. The supernatant serum was stored at −80 °C until the analysis. Total RNA was extracted from serum samples using an miRNeasy Mini Kit (QIAGEN, Hilden, Germany) in accordance with the manufacturer’s instruction. Briefly, 200 µl of serum was lysed in 1000 µl of QIAzol Lysis Reagent. After incubation for 5 minutes, 25 fmol of mirVana miRNA mimic cel-miR-39-3p (Applied Biosystems, Foster City, CA, USA) was added to each sample as an external spiked-in control. Total RNA, including small RNA, was extracted and eluted in 30 µl of RNase-free water.

### Real-time PCR

Serum levels of candidate miRNAs and cel-miR-39 were examined by real-time PCR using TaqMan MicroRNA Assays (Applied Biosystems, Cat. #442797). Every assay IDs are following; let-7b (#2619), miR-15b (#390), -20a (#580), -21 (#397), -29a (#2112), -92a (#431), -122 (#2245), -125b (#449), -141 (#463), -143 (#2249), -145 (#2278), -155 (#2623), -200c (#2300), -221 (#524), -345 (#2186), -423 (#2340), -425 (#1516), -1246 (#CS5I07D), cel-miR-39 (#200). Total RNA (40 ng) was reverse transcribed using TaqMan MicroRNA Reverse Transcription Kit (Applied Biosystems) in a total volume of 20 µl under the following conditions: 16°C for 30 minutes, 42°C for 30 minutes, 85°C for 5 minutes and maintained at 4°C. Real-time PCR was conducted using an MicroRNA Assay Kit and TaqMan Universal Master Mix (Applied Biosystems) and performed in triplicate on the StepOne Plus system (Applied Biosystems) with the following cycling conditions: 95° for 10 minutes, followed by 40 cycles of 95° for 15 seconds and 60° for 1 minute. Cycle threshold (Ct) values were calculated using the StepOne software program, ver. 2.3 (Applied Biosystems). The expression of the miRNAs was normalized to that of cel-miR-39 and determined by the 2^-ΔΔCt^ method. ΔCt was calculated as follows: ΔCt = Ct (miRNA of interest) – Ct (cel-miR-39). ΔΔCt was then calculated using a sample of the calibrator: ΔΔCt = ΔCt (tested sample) - ΔCt (caliblator).

### Statistical analysis

The differences in the serum miRNA levels between responders and non-responders were analyzed using the Mann Whitney-U test. The associations between the clinicopathological factors and the pathological response to nCRT were assessed using the chi-squared two-tailed test or Fisher’s exact test. The independent factors associated with the pathological response were evaluated using a logistic regression analysis. Receiver operating characteristic (ROC) curves with the Youden index were established to determine the threshold of relative quantity in miRNAs to differentiate the pathological response to nCRT. In addition, the area under the curve (AUC) was established using the ROC curve. All of the statistical analyses were performed using the SPSS software program (version 11; IBM, Chicago, IL, USA). *P* values of < 0.05 were considered to indicate statistical significance.

## SUPPLEMENTARY MATERIALS FIGURES AND TABLE


