# Supplementary material for: Serum miR-143 levels predict the pathological response to neoadjuvant chemoradiotherapy in patients with locally advanced rectal cancer
Source: Oncotarget. 2017 Mar 31;8(45):79201–11. doi: 10.18632/oncotarget.16760 (PMC5668032; doi:10.18632/oncotarget.16760)
Supplement: Supplementary file 1 [file oncotarget-08-79201-s001.pdf]

Serum miR-143 levels predict the pathological response to neoadjuvant chemoradiotherapy in patients with locally advanced rectal cancer

Supplementary Materials

Supplementary Table 1: Factors associated with serum miR-143 level

Factor		miR-143 low (*n* = 28)	miR-143 high (*n* = 66)	*P*-value
Age (years)	≤ 60 / > 61	10/18	36/30	0.095
Gender	Male/Female	22/6	41/ 25	0.121
AV-tumor distance (mm)	≤ 40 / > 41	13/15	38/28	0.321
Tumor size (mm)[1]	≤ 40 / > 40	14/14	42/22	0.158
Histological type	well, mod, pap/por	27/1	65/1	0.509
CEA	negative/positive	20/8	49/17	0.778
CA19-9	negative/positive	28/0	60/6	0.174
KRAS[2]	wild type/mutant	18/9	34/31	0.206
cT[3]	2, 3/4	26/2	60/6	1.000
cN[3]	0/1, 2	15/13	40/26	0.527
pT[3]	pCR, is, 1, 2/3, 4b	18/10	32/34	0.160
pN[3]	0/1, 2	23/5	50/16	0.497

[1]The tumor size before CRT was not evaluated by enema examination in two cases.
[2]The KRAS status in two cases was not evaluated.
[3]The clinical and pathological findings were classified according to the TNM classification (7th edition) of the International Union against Cancer (UICC).

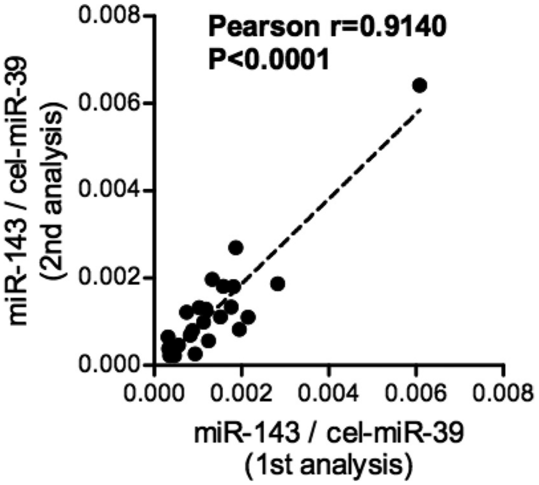

Supplementary Figure 1: A correlation analysis to confirm the reproducibility of the serum miRNA level. RNA extraction and real-time PCR were repeated using serum samples from the same 25 patients, and the serum miR-143 levels of the two experiments were evaluated based on the Pearson correlation coefficients.

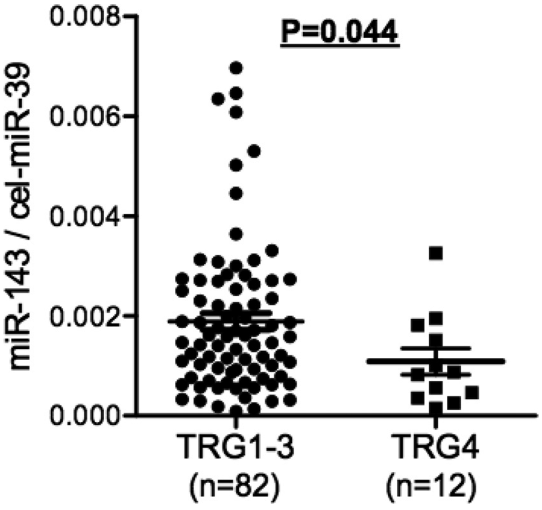

Supplementary Figure 2: A comparison of the serum levels of miR-143 between TRG1-3 and TRG4. The serum miR-143 levels were compared between TRG1-3 tumors ($n = 82$) and TRG4 tumors (pathological complete response, $n = 12$). The Mann Whitney-U test was used.

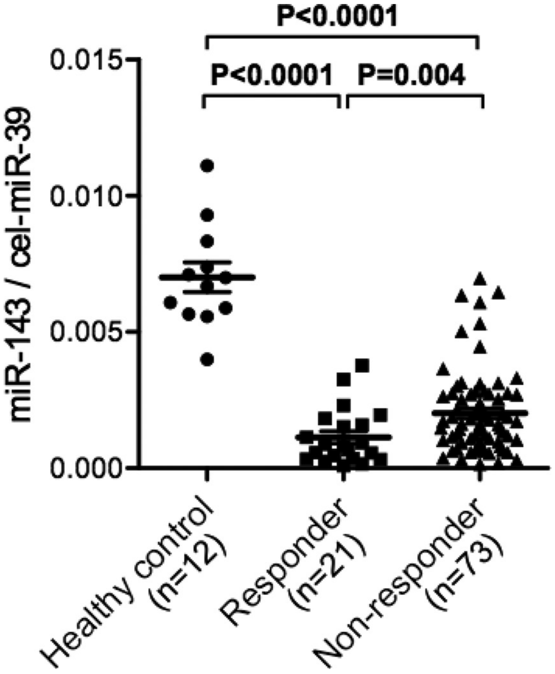

Supplementary Figure 3: A comparison of the serum levels of miR-143 between healthy controls and rectal cancer patients. The serum miR-143 levels were compared between 12 healthy controls and 12 rectal cancer patients (responder: TRG4 and TRG3, non-responder: TRG2 and TRG1). The Mann Whitney-U test was used.

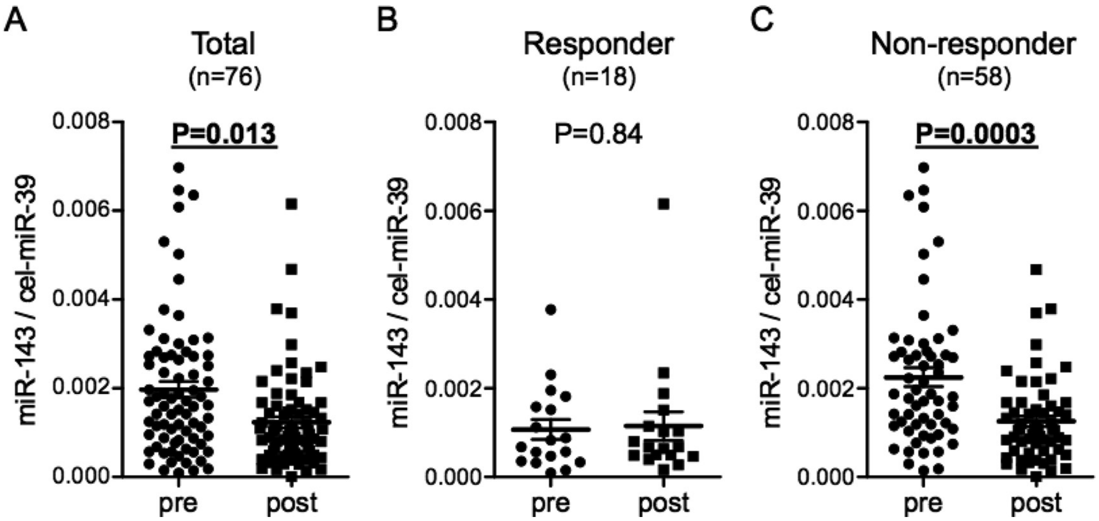

Supplementary Figure 4: A comparison of the serum levels of miR-143 between pre-nCRT and post-nCRT. Serum samples from 76 of 94 patients (18 responders and 58 non-responders) after nCRT (just before surgery) were available for an miR-143 analysis. The serum miR-143 levels were compared between pre- and post-nCRT in the 76 cases (**A**), responders (**B**) and non-responders (**C**), respectively. A paired *t*-test was used. Responders: TRG4 and TRG3, non-responders: TRG2 and TRG1.
